# Whys and Wherefores of Coronary Arterial Positive Remodeling

**DOI:** 10.1161/ATVBAHA.124.321504

**Published:** 2024-10-31

**Authors:** Carlotta Onnis, Renu Virmani, Anna Madra, Valentina Nardi, Rodrigo Salgado, Roberta Montisci, Riccardo Cau, Alberto Boi, Amir Lerman, Carlo N. De Cecco, Peter Libby, Luca Saba

**Affiliations:** 1 Department of Radiology, Azienda Ospedaliero Universitaria, Cagliari, Italy (C.O., R.C., L.S.).; 2 Department of Cardiovascular Pathology, CVPath Institute, Gaithersburg, MD (R.V., A.M.).; 3 Department of Cardiovascular Diseases, Mayo Clinic, Rochester, MN (V.N., A.L.).; 4 Department of Radiology, Antwerp University Hospital and Antwerp University Lier, Belgium (R.S.).; 5 Clinical Cardiology, Department of Medical Science and Public Health, University of Cagliari, Italy (R.M.).; 6 Department of Cardiology, Azienda Ospedaliera Brotzu, Cagliari, Italy (A.B.).; 7 Division of Cardiothoracic Imaging and Biomedical Informatics, Department of Radiology and Imaging Sciences, Emory University, Atlanta, GA (C.N.D.C.).; 8 Division of Cardiovascular Medicine, Brigham and Women’s Hospital, Boston, MA (P.L.).

**Keywords:** atherosclerosis, cardiac imaging techniques, coronary angiography, coronary vessels, diagnostic imaging

## Abstract

Positive remodeling (PR) is an atherosclerotic plaque feature defined as an increase in arterial caliber at the level of an atheroma, in response to increasing plaque burden. The mechanisms that lead to its formation are incompletely understood, but its role in coronary atherosclerosis has major clinical implications. Indeed, plaques with PR have elevated risk of provoking acute cardiac events. Hence, PR figures among the high-risk plaque features that cardiac imaging studies should report. This review aims to provide an overview of the current literature on coronary PR. It outlines the pathophysiology of PR, the different techniques used to assess its presence, and the imaging findings associated to PR, on both noninvasive and invasive studies. This review also summarizes clinical observations, trials, and studies, focused on the impact of PR on clinical outcome.

**Highlights**

- The coronary arterial tree undergoes dynamic changes over time in response to various factors, with atherosclerosis driving structural and hemodynamic alterations in the vessel wall, leading to either negative remodeling or positive remodeling (PR).
- PR, while preserving the vessel lumen, increases the risk of plaque rupture and acute coronary syndrome, making it a critical feature in coronary artery disease assessment.
- PR invasive assessment can occur through intravascular ultrasound and optical coherence tomography, both of which provide valuable insights into plaque characteristics; intravascular ultrasound is particularly recommended for assessing high-risk plaque features and correlating remodeling index values with clinical outcomes.
- Noninvasive methods such as coronary computed tomography angiography provide significant advantages for screening and monitoring, correlating well with intravascular ultrasound findings; advanced coronary computed tomography angiography technologies enhance spatial resolution, allowing detailed plaque characterization.
- PR serves as a predictor of plaque vulnerability, increased risk of rupture, and worse outcome. Its evaluation with coronary computed tomography angiography may enhance risk assessment for nonobstructive lesions, highlighting its role in risk stratification. Further studies are needed to clarify how the presence of this feature may impact patient management and how PR changes in response to treatment.

The coronary arterial tree changes with time in response to multiple factors. The coronary vessel wall is a trilaminar structure, composed of endothelium, intima, which contains extracellular matrix and scattered smooth muscle cells (SMCs), the tunica media with multiple layers of SMCs, and the outer adventia.^1^ Alterations in the structure and biological characteristics of these layers profoundly affect the clinical manifestations of atherosclerosis. This progressive disease characterized by deposition and accumulation of lipids accounts for an increasing global burden of cardiovascular disease (CVD) and coronary artery disease (CAD).^2^ Atherosclerosis leads to progressive morphological changes, ranging from intimal thickening, formation of early atheroma, progression to fibroatheroma (with a thick or thin fibrous cap), and calcification.^3^ Atherosclerosis-driven morphological changes induce hemodynamic changes of the coronary flow, which, concurrently, activate a response of the coronary vasculature that leads to more structural changes. This cyclic process of arterial remodeling can occur in an outward or inward direction, called positive or negative remodeling, respectively. These different directions of progression that the plaque has critical clinical implications.

Coronary vessels that remodel negatively promote luminal narrowing, thus exacerbating stenosis. However, in positive remodeling (PR), although it preserves the luminal caliber, the atherosclerotic plaque becomes more prone to rupture and erosion, mechanisms that provoke acute coronary syndrome (ACS). This finding has progressively led the literature toward a more in-depth evaluation of PR, with inclusion of PR being included as a high-risk plaque (HRP) feature evaluated on diagnostic tests as coronary computed tomography angiography (CCTA).^4^

PR is defined by evaluating the change in outer vessel diameter, representing the external elastic lamina, at the plaque location when compared with a normal-appearing site, taken as reference diameter. Remodeling evaluation can be performed through visual assessment of cross-sectional images, longitudinal reconstructions of the vessel, or through quantitative assessment. The latter uses the remodeling index (RI), a categorical parameter that indicates direction of remodeling, often defined as the ratio between the vessel diameter at the level of the lesion and a chosen reference diameter. Specific cutoff to define RI depends on the technique used, for example, computed tomography (CT)–derived RI is considered positive when the diameter at the plaque site is at least 10% larger than the one at the reference site.^5^

This review summarizes the current understanding and literature on coronary artery remodeling. The aim is to discuss its pathophysiology, with a focus on the 2 directions, outward and inward, that remodeling can follow; highlight the major studies in literature that evaluate its clinical implications; and give an overview of the current diagnostic techniques that allow remodeling assessment.

## How Does It Form?

Glagov et al^6^ showed in 1987 that PR prevents lumen narrowing during atherosclerosis progression until the plaque burden reaches a certain threshold, around 40%, after which narrowing starts to occur. The mechanism behind this process is multifactorial. Pathophysiology of coronary artery remodeling involves endothelial dysfunction, inflammation, lipid accumulation, and platelet activation. How these factors interact can shift the remodeling toward an outward or inward direction.

Positive remodeling and negative remodeling are thought to be related to the type of flow change that can occur, and an important role is played by the plaque-free wall. Following changes in the blood flow and circumferential stretch, induced by increasing plaque burden, functional endothelial cells—mostly on the plaque-free vessel side—are stimulated to produce and release growth factors, proteases, and proinflammatory cytokines.^7^ Wentzel et al^8^ have shown that the plaque-free wall was associated with vascular remodeling in a 3-year follow-up and that it correlated inversely with plaque burden. In case of sustained flow, endothelial cells of the vessel wall free of plaque adapt to the increased shear stress and release stretch-induced fibroblast growth factor-2, responsible for SMC proliferation and migration, MMPs (matrix metalloproteinases), responsible for extracellular matrix and elastic lamina degradation, and NO, which can activate MMPs.^9^ Accumulation of the SMCs against a disrupted elastic lamina can facilitate an outward remodeling and also lead to increased vulnerability. Some experimental data implicate MMP-3 in expansive remodeling during atherogenesis in mice.^10^ Conversely, negative remodeling is associated with a more circumferential distribution of plaque, which does not leave any vessel wall free of plaque, stimulating an inward direction of remodeling. Moreover, during a low-flow state, endothelial cells release transforming growth factor-β and platelet-derived growth factor, which can promote the proliferation and migration of SMCs toward the lumen and collagen production, mediating negative remodeling. Overall matrix degradation depends on the balance between MMPs and their inhibitors. As the ratio between MMP and MMP inhibitor increases, matrix degradation and PR will be facilitated. Alternatively, a decreased ratio contributes to greater collagen turnover, facilitating negative remodeling.^7^

Moreover, accumulation and activation of oxidized LDL (low-density lipoprotein) plays a crucial role in plaque vulnerability, as suggested by Ichikawa et al.^11^ The authors found that high levels of MDA-LDL, a form of oxidized lipoprotein, are significantly associated with the presence of HRP features and are an independent predictor of cardiovascular events, even in patients treated with statin. The mechanism by which LDL acts involves multiple factors: activation of LDL receptors in endothelial cells contributes to MMP activation,^12^ and accumulation of oxidized LDL damages SMCs and induces their apoptosis.^13^ Both these processes favor plaque instability.^14^ Similarly, McGarrah et al,^15^ in the PROMISE trial (Prospective Multicenter Imaging Study for Evaluation of Chest Pain), showed that greater concentration of medium and large HDLs (high-density lipoproteins) was associated with decreased risk of HRP, as opposed to LDL. Medium HDL associated inversely with major adverse cardiovascular events (MACE). Although the potential protective role of HDL has come under considerable doubt, the pathogenic effects of LDL on the occurrence of future ACS events remain indisputable.^16^ Additionally, it has been shown that PR coronary plaques have greater lipid accumulation, when compared with negative remodeled ones, and this may contribute to the greater vulnerability associated with PR, providing a possible explanation of the paradoxical effect seen in PR: protective effect due to lesser vessel stenosis and harmful effect due to greater plaque vulnerability.^17^ The factors balancing compensatory outward remodeling, which prevents luminal stenosis in early atherogenesis, and excessive outward remodeling, which increases vulnerability, are not fully understood. However, low shear stress has shown to promote internal elastic lamina degradation and intense inflammation, which facilitates lipid accumulation and excessive vascular wall expansion.^18^ Indeed, high lipid content might further stimulate MMP production, allowing excessive PR at sites made vulnerable by greater lipid core. Conversely, in compensatory PR, the lipid core is smaller, surrounded by a thick fibrous cap, preventing fast plaque progression, acute plaque events, and symptomatic luminal narrowing.

Inflammation plays an important role in plaque remodeling as well and strictly correlates with the integrity of the endothelium.^19,20^ Endothelial dysfunction is indeed associated with altered vascular permeability, which is necessary for inflammatory cell migration, as well as platelet deposition and subsequent activation.^19^ When endothelial dysfunction occurs, the endothelial monolayer is denudated, with loss of cellular junctions; this phenomenon is one of the multiple factors that can incite inflammation in atherosclerosis.^21,22^ Recruitment of macrophages and monocytes induced by proinflammatory cytokines seems to be shear related, and it potentiates MMP activation.^23–25^ As suggested by Varnava et al,^17^ remodeling correlates with inflammatory cell infiltrate at the adventitia opposite the plaque, and the greater inflammatory infiltrate was seen among PR plaques, indicating that outward remodeling might begin at the plaque-free arc of the vessel, stimulated by the presence of inflammatory cells. Another indicator of the key role of inflammation is suggested by Zhao et al.^26^ The authors investigated endothelial cell response to shear stress and showed that increased shear stress corresponds to an increased proinflammatory endothelial-to-mesenchymal transition, via regulation of gene expression, such as the nuclear factor-κB signaling pathway. These findings draw attention to the role of genetic factors in vascular remodeling. Studies have shown that specific allelic variations are associated with the expression or loss of function of genes able to modify the course of atherogenesis.^27,28^ For example, loss of function of gene *SMAD3* in SMCs was associated with greater PR, and the presence of knockdown *RGS5* gene exacerbated vascular remodeling, while its overexpression attenuated the remodeling process.^29,30^ Understanding the role of gene regulators in coronary remodeling represents a future research focus for the development of targeted therapy.

**Figure 1.**
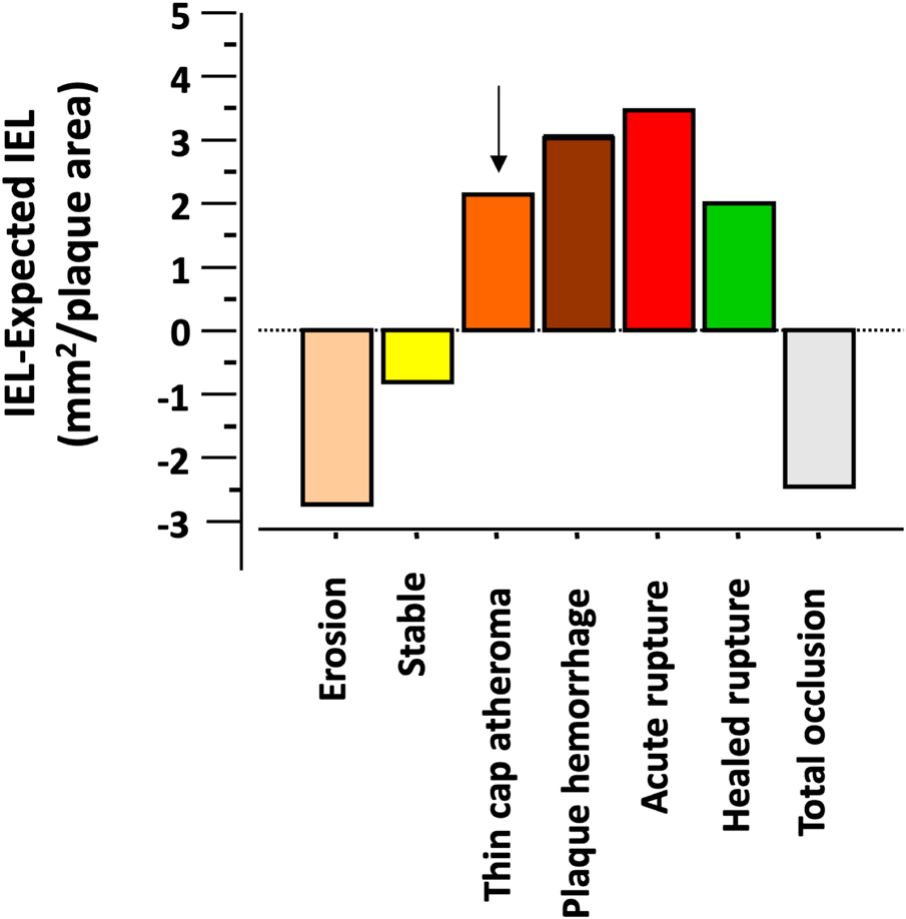
**Remodeling by plaque type.** The internal elastic lamina (IEL) increase, adjusted for plaque area, is plotted by plaque type. Plaque hemorrhage, including plaque rupture, showed significantly greater remodeling than fibrous plaque (stable; *P*=0.03) and total occlusion (*P*=0.03); the test used is ANOVA. Data derived from Burke et al.^31^

**Figure 2.**
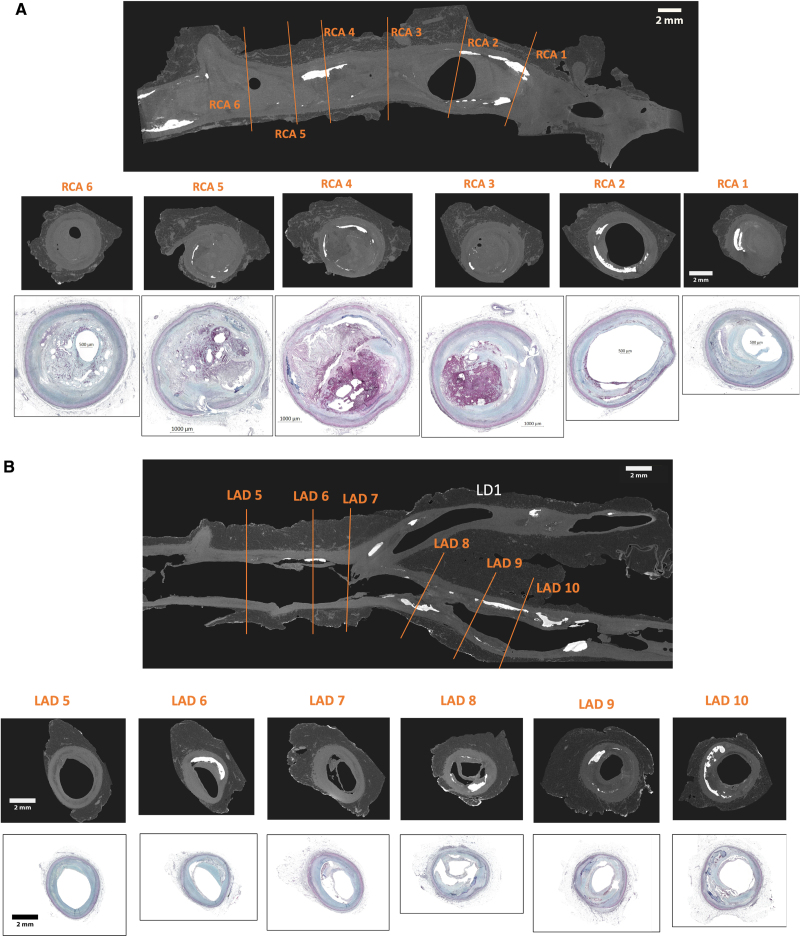
**A 55-year-old male patient who died suddenly without any medical history.** Autopsy, micro-computed tomography (micro-CT) after coronary perfusion fixation, and histological findings at 3- to 4-mm intervals (the coronary arteries were removed from the heart, perfusion fixed, and then the specimen was imaged with X-ray micro-CT, Nikon XTH 225 ST PE1621 EHS system using a tungsten target at 95 keV and 89 µA with 1200–1800 projections of 500 ms each resulting in a 20- to 30-minute scan with a resolution ranging between 18.4 and 23.1 µm). The scans were reconstructed using Core Imaging Library 23.1.0 and postprocessed in ImageJ/Fiji 1.54h and 3D Slicer 5.4.0. **A**, Plaque rupture at the proximal right coronary artery (RCA). The proximal end is on the right, and the distal segment is on the left. The orange lines indicate where the sections were taken showing positive remodeling, and the corresponding histological sections show the area of plaque rupture in RCA 4. Note this section is the most remodeled with mild calcification and a large necrotic core. Section labeled RCA 5 shows total occlusion and RCA 6 shows organized thrombus with severe narrowing of the lumen. **B**, Thin-cap fibroatheroma (TCFA) of the left anterior descending (LAD). The midsection of the LAD is shown with the left diagonal coming off the LAD (above). The orange lines indicate where the sections were taken showing positive remodeling, with maximum remodeling in LAD 10, and the corresponding histological sections show the area of TCFA in sections LAD 8 to LAD 10. Images RCA 4 and LAD 10: data derived from Jinnouchi et al.^32^ LD indicates left diagonal.

Given the multifocal, multifactorial, systemic nature of atherosclerosis, different types of remodeling can coexist in the same patient at a single point in time, and changes in local factors, such as shear stress and inflammation, can alter the local remodeling response over time. Even though it is thought that the first arterial change occurring in response to atherosclerosis is compensatory enlargement of the outer vessel wall and lumen, consisting in PR, from early fibroatheroma, to late stable or unstable plaque, vascular remodeling should be seen as a continuum, influenced by many factors. Burke et al^31^ reported coronary remodeling in 36 hearts from individuals who had died of severe CAD, after perfusion fixation at 100 mm Hg. Plaques were positively remodeled in lesions with fibroatheroma, thin-cap fibroatheroma, plaque hemorrhage, plaque rupture, and healed plaque rupture (Figure 1); of these, plaque hemorrhage and plaque rupture showed the greatest remodeling. Whereas plaques with erosion, fibrous plaques, and total occlusions showed negative remodeling. The study also showed that after adjusting for age, sex, and distance from the coronary ostia, plaque components of macrophage infiltration, percent lipid core, percent fibrous and lipid core calcification, and medial atrophy were strongly associated with remodeling. However, percent fibrous tissue and adventitial thickness were negatively associated with remodeling (Figure 2).

## How Do We Image It?

### Invasive Techniques

**Figure 3.**
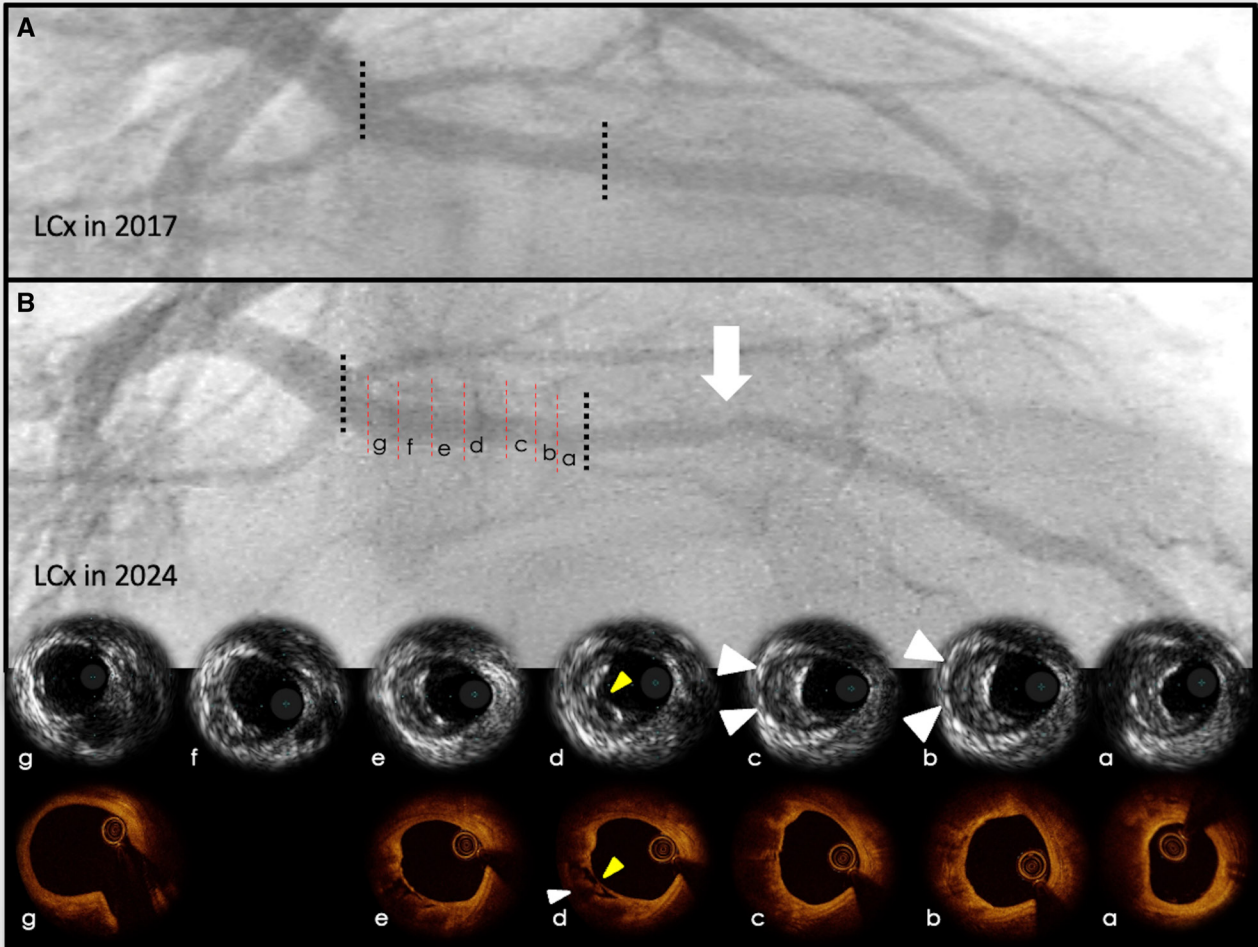
**Male patient with multiple cardiovascular risk factors at baseline and follow-up. A**, A 41-year-old male patient with multiple cardiovascular risk factors and mild atherosclerosis of the left circumflex (LCx). **B**, Seven years later, the patient presented with unstable angina. Percutaneous coronary intervention revealed severe stenosis of the mid portion of LCx (white arrow). Intravascular ultrasound images of the segment proximal to the stenosis are shown above; note the presence of a nonobstructive atherosclerotic plaque with positive remodeling (white arrowheads) and ulceration (yellow arrowhead). Optical coherence tomography, performed at the same level, is shown below; note the presence of thin-cap fibroatheroma (yellow arrowhead) and necrotic core (white arrowhead).

Detection of PR can occur invasively and noninvasively. The first studies on plaque characterization were conducted invasively, with intravascular ultrasound (IVUS), after assessment of its accurate correlation with histology.^33^ Thereafter, the reference standards have become IVUS and optical coherence tomography (OCT), both well-established techniques (Figure 3). IVUS during percutaneous intervention has received a class IIa recommendation in the European Society of Cardiology 2018 guidelines on myocardial revascularization and in the 2011 and 2021 American Heart Association/Society for Cardiovascular Angiography and Intervention guidelines for coronary artery revascularization, particularly for left main and ostial stenosis assessment, stent optimization, and reevaluation.^34–36^ They allow the detection and characterization of HRP features, with analysis of the obtained images providing quantification, through calculation of the RI. Histopathologic and IVUS analyses have defined remodeling of an atherosclerotic lesion by comparing absolute lesion-level external elastic lamina cross-sectional measurements to a reference, nondiseased, segment, proximal or distal to the lesion, at a single point in time. The ratio of the resulting measurements defines the RI: values >1.05 indicate PR, whereas values <0.95 suggest negative remodeling, and values between 0.95 and 1.05 indicate the absence of remodeling.^37^ The remodeling response of the coronary vessel is a continuous variable, and RI represents its categorical counterpart, indicating the direction of the phenomenon. Despite being an indirect measurement, assessed at a single, static point of time, of a dynamic process, RI has been shown to remain relatively constant over time, and PR lesions continued to undergo the process.^38^ Moreover, measurement of RI pre-intervention has shown to correlate with clinical outcome after stenting.^39^ An RI >1.05 before intervention was associated with a higher risk of intimal hyperplasia in response to stenting and unfavorable clinical outcome. These results suggest that remodeling evaluation with IVUS, prior intervention, may help stratify lesions at greater risk and call for tailored management.^39^

While conventional IVUS evaluates plaque in gray scale, postprocessing with frequency domain analysis of IVUS radiofrequency data allows virtual histology of the plaque. This technique, even though not widely clinically used, provides better characterization of the plaque components.^40,41^ Hong et al^42^ used virtual histology to evaluate the relation between remodeling and other plaque characteristics; they showed that patients with PR coronaries have plaques with greater necrotic core, less fibrotic component, and more thin-cap fibroatheroma, when compared with those with negative remodeling.

Resolution of novel IVUS systems, which use higher frequencies (40–60 MHz), can reach 22 µm, approaching that of OCT, the technique with the highest spatial resolution.^43^ Achievement of such high resolution improves the detection of high-risk features. OCT is a technique that uses optical echoes of near-infrared light and provides high-resolution images (10–15 µm). As such, plaque visualization and characterization are extremely detailed. However, comparison studies have shown that data obtained with this technique correlate with data derived from IVUS.^44,45^ However, as OCT penetration is lower than that of IVUS (1–2 versus 4–8 mm), visualization of all layers of the coronary wall, including the external elastic lamina, is limited, making it less suitable for PR assessment.

### Noninvasive Techniques

**Figure 4.**
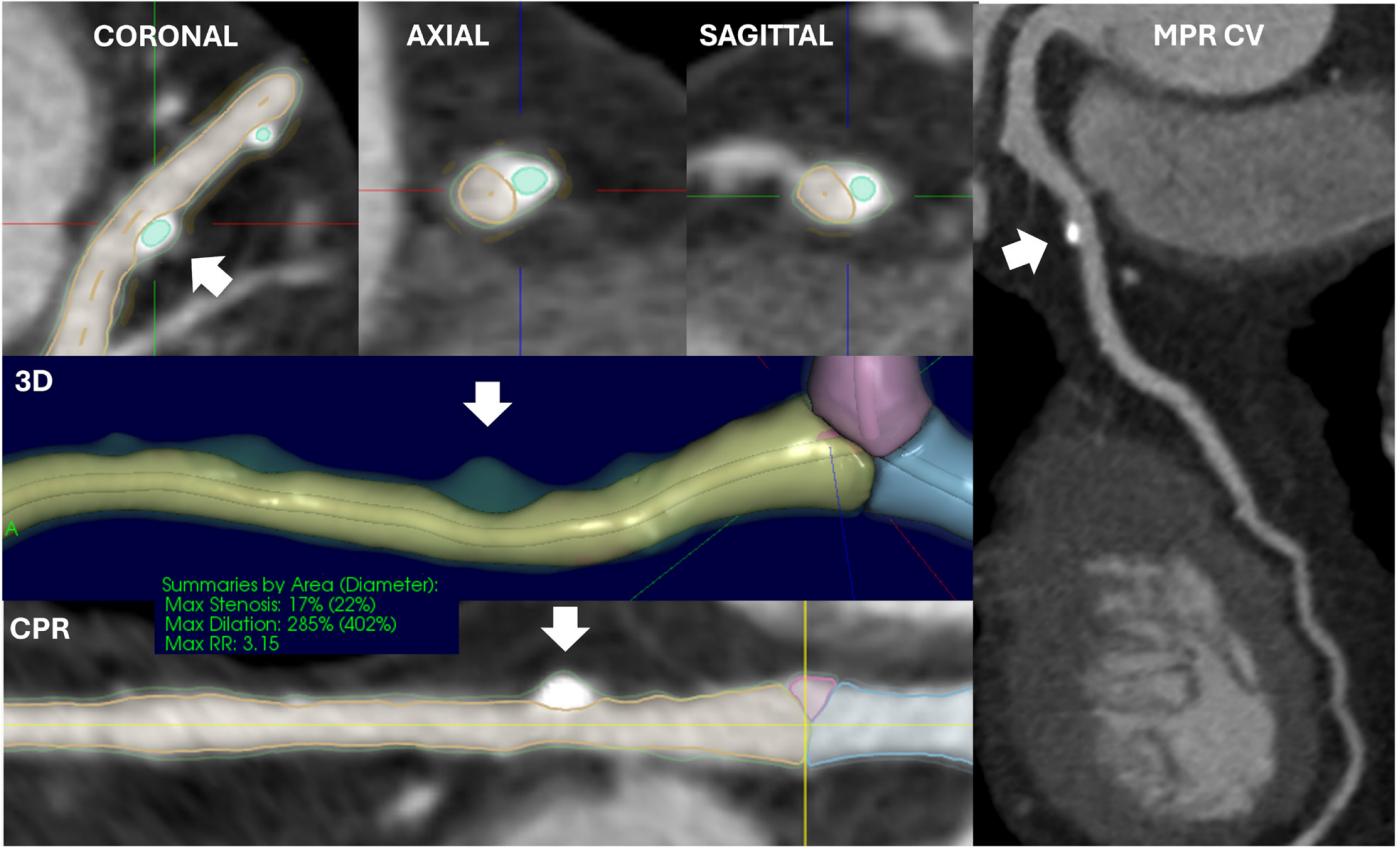
**Coronary computed tomography angiography images of a 68-year-old male patient presenting with chest pain to the emergency department and multiple risk factors for coronary artery disease.** Images show a calcified atherosclerotic plaque in the proximal left anterior descending (LAD) with positive remodeling with mild stenosis, ≈30% (white arrow). Coronary plaque analysis was performed with a semiautomatic software (vascuCAP; Elucid Bioimaging, Wenham, MA). Software analysis provides coronal, axial, and sagittal views, as well as 3-dimensional (3D) and curved planar reformation (CPR) straightened views. Software output includes remodeling ratio (RR), percent dilation, and percent stenosis, which in this case were 3.15, 285%, and 17%, respectively. Curved multiplanar reformation of the LAD is shown on the right. Max indicates maximum; and MPR CV, multiplanar reformation curved view.

Although invasive techniques represent the reference standard for the evaluation of HRP, noninvasive techniques offer considerable advantages for screening, monitoring, and rapid detection of HRP. CCTA represents the main noninvasive imaging modality for the detection of PR and correlates with virtual histology and IVUS findings.^46^ Conventional CT scanners have a spatial resolution of 0.4 mm. Excitingly, novel scanners such as photon-counting CT can substantially improve this resolution.^47,48^ These techniques provide detailed images and allow plaque characterization, establishing CCTA as a well-validated method, as illustrated in the Coronary Artery Disease–Reporting and Data System, version 2.0 (updated version; Figure 4).^49^ Studies have assessed the accuracy of CT for detecting PR as compared with established reference IVUS standard. Gauss et al^50^ have shown that CCTA typically overestimates the RI, thus a higher threshold (1.1 for CCTA versus 1.05 for IVUS) of this index has been applied to CCTA for optimal classification. Similarly, Boogers et al^51^ demonstrated the feasibility of plaque quantification on CCTA using a coregistration algorithm to compare CCTA and IVUS. Thus, in CCTA scans, the definition of PR is an RI >1.1.^4^ The relationship between quantitative plaque measurements, such as RI, and qualitative HRP features on CCTA has been investigated within the ROMICAT-II trial (Rule Out Myocardial Ischemia/Infarction by Computer Assisted Tomography).^52^ Authors demonstrated an association between quantitative measurements and the presence of HRP features identified by expert readers and concluded that quantitative coronary plaque characterization correlates with qualitative analysis. A significant advantage of CCTA when compared with IVUS and OCT is the 3-dimensional coronary visualization. While invasive techniques only acquire axial cross-sectional images, CCTA provides cardiac imagers with a processed data set, allowing multiplanar reconstruction and curved planar reformations of the coronary tree, which may be helpful when assessing the vessel. However, these features come at the expense of necessary radiation exposure. Nevertheless, current CCTA acquisition can involve relatively low radiation exposure, rendering it an acceptable modality for screening but also for follow-up and treatment response monitoring of potential plaque vulnerability. Moreover, with the advent of artificial intelligence, measurement of RI in a semiautomated or fully automated fashion has simplified and made faster quantitative plaque assessment.^53^

Other noninvasive techniques that can directly or indirectly indicate the presence of PR are cardiac magnetic resonance and positron emission tomography. Cardiac magnetic resonance can detect coronary remodeling with specific black-blood coronary wall magnetic resonance images, but results still require clinical correlation.^54,55^ As for positron emission tomography, ^18^F-NaF (^18^F-sodium fluoride) positron emission tomography seems to provide an indirect indication of the presence of PR, as shown by Joshi et al.^56^ Authors observed that lesions with increased uptake, suggestive of higher grade of inflammation and metabolic activity, were associated with greater PR on IVUS, highlighting the higher grade of inflammation that occurs in HRP. Further evaluation is being performed in the PREFFIR study (Prediction of Recurrent Events With ^18^F-Fluoride), a multicenter study focused on ^18^F-NaF as a marker of plaque vulnerability (https://www.clinicaltrials.gov; unique identifier: NCT02278211).

## How Does It Impact Clinical Management?

PR can predict plaque vulnerability, indicating higher risk of rupture.^4^ Patients presenting with atherosclerotic lesions containing high-risk features, such as PR, have a greater risk of future events; thus, many studies have focused on the clinical implications and outcome correlation of PR (Table). Overall, the prevalence of PR was higher in patients with high atherosclerotic CVD risk score; among those patients, greater prevalence of hypertension, diabetes, smoking, and male sex was also noted, as well as advanced age and increasing body mass index.^57,58^ Prevalence of PR was greater with increasing degree of stenosis and significance of CAD (≥50% stenosis), which was also associated with greater frequency of dyslipidemia and smoking.^59,60^ Among patients with acute myocardial infarction undergoing preprocedural and postprocedural IVUS, the presence of single plaque rupture, multiple plaque ruptures, and thrombus was more frequent among patients with PR; also plaque prolapse, defined as tissue extruding the stent strut post-procedure, and postprocedural myonecrosis, indicated by cardiac-specific troponin I, were observed more frequently in lesions with PR, when compared with those with negative remodeling. These results might suggest a more severe presentation of acute myocardial infarction in patients with PR.^61^

**Table.**
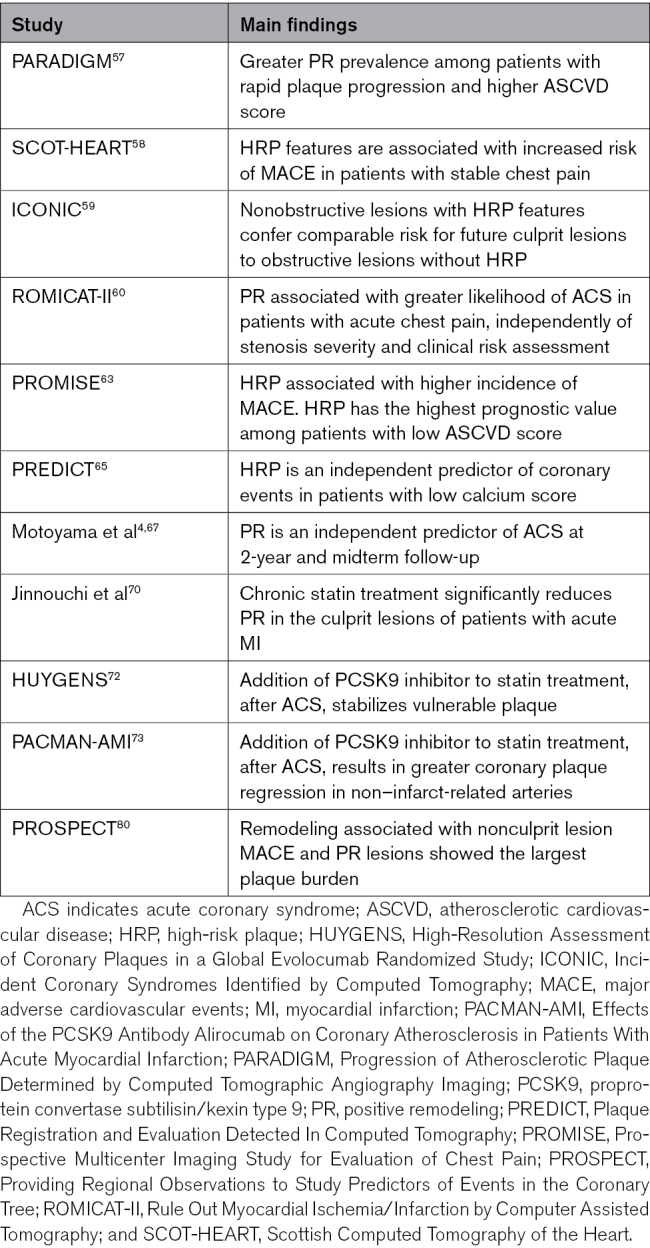
Clinical Implications of PR

CCTA studies have shown that greater PR was associated with culprit lesions, rather than stable ones.^62^ Moreover, in the PARADIGM study (Progression of Atherosclerotic Plaque Determined by Computed Tomographic Angiography Imaging), which evaluated the association between serial CCTA and clinical presentation, PR was found to have greater prevalence among patients with rapid plaque progression and high atherosclerotic CVD risk score, at baseline CCTA. Among this group of patients, the development of new PR was also greater.^57^ These findings suggest that PR is not only associated with high cardiovascular risk at baseline but also that its development is accelerated among these patients over time. Additionally, the ICONIC study (Incident Coronary Syndromes Identified by Computed Tomography) has observed that nonobstructive HRP-positive lesions conferred similar risk to that of obstructive HRP negative ones and that the absolute number of HRP is greater among nonobstructive, nonsevere lesions, when compared with obstructive lesions.^59^ In patients with acute chest pain, PR associates with increased likelihood of ACS, independently of stenosis severity and clinical risk assessment, as seen in the ROMICAT-II trial.^60^

Moreover, PR negatively impacts the outcome of patients presenting with stable chest pain: as highlighted by the SCOT-HEART study (Scottish Computed Tomography of the Heart), adverse plaque characteristics are also associated with an increased risk of MACE, specifically coronary heart disease death and nonfatal myocardial infarction.^58^ Similarly, the PROMISE trial evaluated stable, symptomatic outpatients and showed that HRP, found on CCTA, was linked to a higher incidence of MACE, strongly predicting MACE, especially in women and young patients.^63^ Another finding of the PROMISE trial is that, when comparing the prognostic value across categories of clinical cardiovascular risk, HRP has the highest prognostic value in patients with low atherosclerotic CVD risk. This observation suggests that HRP detection can improve risk stratification in symptomatic low-risk patients, which may be otherwise left untreated or treated less aggressively.^64^ Similarly, Yamamoto et al,^65^ in their post hoc analysis of the PREDICT registry (Plaque Registration and Evaluation Detected In Computed Tomography), found that HRP independently predicts coronary events in patients with suspected CAD and low coronary calcium score, indicating the additional predictive value of HRP. Moreover, in patients with low-risk stable chest pain and nonobstructive CAD, the addition of adverse plaque features (≥2 HRPs) to traditional risk prediction models, such as atherosclerotic CVD risk score, degree of stenosis, coronary calcium score, and segment involvement score, resulted in a significant improvement of model fit and discrimination of future clinical events, underlying that HRP has incremental value over total plaque burden and traditional risk factors.^66^ Motoyama et al^4,67^ observed that PR is an independent predictor of ACS both at 2-year follow-up and midterm follow-up (median, 4.1 years). A recent meta-analysis has shown that coronary plaque characteristics, including PR, assessed by either CCTA or IVUS, consistently predict MACE, at a per-lesion and per-patient level.^68^ The authors also suggested that, even though guidelines define HRP as the presence of ≥2 features, the overall event risk prediction can be achieved with better accuracy and sensitivity, with only a small decrease in specificity, when defining HRP as the presence of at least 1 feature.^68^

The presence in the literature of these studies and trials, focusing on the association between CCTA-derived PR and outcome, has led to its inclusion among the HRP features stated in the first Coronary Artery Disease–Reporting and Data System and its updated version (version 2.0).^49,69^ Detection of HRP on CCTA, defined as the presence of at least 2 high-risk features, as indicated in Coronary Artery Disease–Reporting and Data System, version 2.0, can impact further management. Regardless of clinical presentation (acute versus stable chest pain), the identification of HRP, including PR, should prompt the need for more aggressive preventive treatment, even in the absence of severe stenosis. The importance of statin treatment has been stressed by Jinnouchi et al^70^ and by Suzuki et al,^71^ who showed that chronic statin treatment significantly reduces PR via the inhibition of plaque progression in culprit lesions of patients with myocardial infarction and known CAD, respectively. More recently, the trials HUYGENS (High-Resolution Assessment of Coronary Plaques in a Global Evolocumab Randomized Study)^72^ and PACMAN-AMI (Effects of the PCSK9 Antibody Alirocumab on Coronary Atherosclerosis in Patients With Acute Myocardial Infarction)^73^ showed that the addition of PCSK9 inhibitors to high-intensity statin therapy favorably modifies coronary plaque in high-risk patients, such as those with recent ACS. Additionally, the detection of HRP or greater vessel complexity on CCTA may be helpful for selecting those patients who would benefit from IVUS evaluation during percutaneous coronary intervention. In fact, studies have shown that the use of IVUS for evaluation of complex lesions was associated with a lower risk of medium-term mortality and target vessel revascularization.^74,75^

## Limitations

Despite the promising results of some studies in the literature, some authors have raised questions regarding the role of PR as a valuable risk factor and marker of future MACE.^76,77^ Lee et al^78^ have shown that, even though plaque progression occurred more frequently among HRP lesions, the presence of an HRP itself was not a significant factor in the prediction of lesion progression to obstructive. Similarly, in a previous study, Lee et al^79^ observed that HRP added little value in the prediction of rapid plaque progression on a per-patient basis. PR, as well as the other HRP features, might represent a coexisting marker of atherosclerosis, more frequently seen in advanced stages, rather than a direct marker of plaque progression. Additionally, in the PROSPECT study (Providing Regional Observations to Study Predictors of Events in the Coronary Tree), Inaba et al^80^ emphasized the concept of bidirectional remodeling as a marker of future nonculprit lesion MACE, rather than PR alone. They investigated the correlation between remodeling patterns and clinical outcome among patients with ACS evaluated with IVUS and observed that MACE occurred more frequently in the positively and negatively remodeled lesions, compared with the intermediate ones. Hence, they questioned the role of PR alone and suggested that both directions of remodeling might be associated with future events.^80^ However, in the same study, greater plaque burden, a known predictor of MACE, was observed among PR lesions, when compared with negative and intermediate remodeling, and additional IVUS studies have suggested the correlation between PR, greater plaque burden, and the occurrence of future ACS.^37,81,82^

## Conclusions

Clinical observations have highlighted the importance of PR and its relevant role on vulnerability. Therefore, the presence of PR, as well as other HRP features, could play a role in risk assessment and decision-making, especially when reclassifying the risk of nonobstructive lesions that might be otherwise underappreciated. The possibility offered by CCTA to evaluate nonobstructive, mild stenotic lesions, and the presence of HRP in a noninvasive, fast fashion is promising. Effort should be made to further validate PR assessment via CCTA against invasive gold standard represented by IVUS. Moreover, further studies are needed on the use of novel CT techniques, such as photon-counting CT, for PR assessment.

In conclusion, PR holds great potential as a marker of atherosclerotic progression, and guidelines recommend reporting the presence of this feature, in the setting of HRP modifier of Coronary Artery Disease–Reporting and Data System, version 2.0, to indicate greater plaque burden and vulnerability. However, further studies are needed to clarify how the presence of this feature may impact patient management and how PR changes in response to treatment.

## Article Information

### Sources of Funding

None.

### Disclosures

None.
